# Development and Application of Eddy Current Sensor Arrays for Process Integrated Inspection of Carbon Fibre Preforms

**DOI:** 10.3390/s18010004

**Published:** 2017-12-21

**Authors:** Dietrich Berger, Gisela Lanza

**Affiliations:** Karlsruhe Institute of Technology (KIT), wbk Institute of Production Science, 76131 Karlsruhe, Germany; gisela.lanza@kit.edu

**Keywords:** carbon fibre preforms, eddy current testing, in-process quality inspection

## Abstract

This publication presents the realisation of a sensor concept, which is based on eddy current testing, to detect textile defects during preforming of semi-finished carbon fibre parts. The presented system has the potential for 100% control of manufactured carbon fibre based components, allowing the immediate exclusion of defective parts from further process steps. The core innovation of this system is given by the high degree of process integration, which has not been implemented in the state of the art. The publication presents the functional principle of the sensor that is based on half-transmission probes as well as the signals that can be gained by its application. Furthermore, a method to determine the optimum sensor resolution is presented as well as the sensor housing and its integration in the preforming process.

## 1. Introduction

The use of carbon fibre reinforced plastics (CFRP) in automotive applications has different economical, ecological and functional benefits. To increase the potential of this material type, its industrial production, however, faces challenges such as the reduction of production costs or the handling of process variations that result in quality critical material flaws. At the same time, overall production volumes of CFRP components must be increased to significantly reduce automotive pollutant emissions that are bound to the vehicle’s mass [1,2,3].

An approach to face these challenges involves the integration of methods for production integrated quality control. In the case of Resin Transfer Moulding (RTM), it has been shown that production costs can be reduced significantly by controlling the quality of so-called carbon fibre preforms prior to the infiltration step [4]. The reason is that flaws such as textile wrinkles of fibre tow misorientations mostly occur during preforming of semi-finished carbon fibre textiles. When excluding defective parts from the following process steps, unnecessary costly value added can be avoided. Therefore, it is beneficial to pursue a high level of integration for these methods that are capable of testing or measuring quality critical characteristics within the tact time of preforming. The highest degree of integration is referred to as “in-process integration” which allows the non-destructive quality evaluation of each produced component during the production process [5].

Eddy current testing has proven that it can detect the major part of industry relevant flaws, however, with a small degree of production integration. Therefore, the state of the art in eddy current testing is presented, showing the potential and necessity of in-process eddy current sensors for the quality control of carbon fibre preforms.

Eddy current testing of carbon fibre structures is based on the anisotropic conductivity of carbon fibre textiles, both cured and as a semi-finished part. Proper fibre tow orientations have a crucial impact on part strength and durability, as they are the load bearing component within carbon fibre reinforced plastics. Common production tolerances for fibre tow orientations are within 3°–5° individually depending on the laminate structure [6,7].

**Figure 1:**
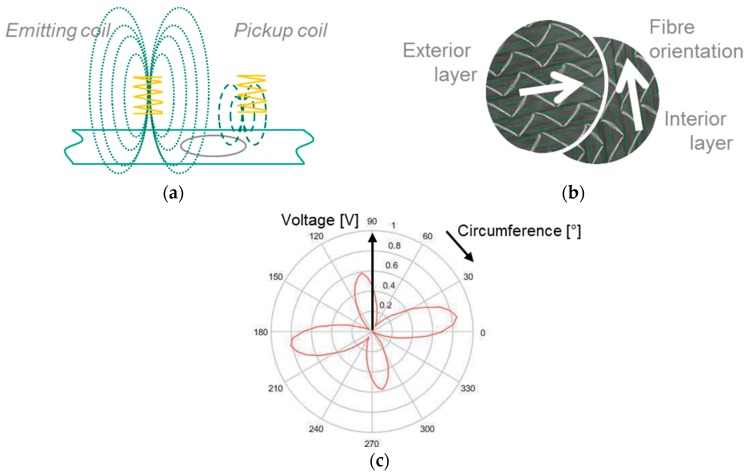
(**a**) Hal-transmission probe arrangement [9]; (**b**) two-layer material specimen; and (**c**) anisotropically induced voltage measured in pickup coil.

Sensor arrangements that are common when testing carbon fibre structures via eddy current were presented and evaluated by Schmidt, whose conclusion is that half-transmission probes are best used for the described purpose [8]. This probe type consists of an emitting and a pickup coil. In this arrangement, the emitting coil is connected to a high frequent voltage that is generating a magnetic field. Due to this magnetic field, eddy currents are caused inside the inspected carbon fibres and conducted anisotropically. The measurement of the induced voltage in the pickup coil leads to the indirect determination of fibre tow orientations and stack structure. The described relationships are depicted in Figure 1a–c, respectively, and were obtained experimentally by rotating a half-transmission probe arrangement above a two-layer material specimen, each consisting of a unidirectional textile with a relative rotation of 90° to each other. The measured voltages are plotted in a polar diagram.

Lange and Mook present a concept where a rotational probe, consisting of two electric coils, is mechanically moved above the surface of a cured specimen. The rotation of the probe above predefined regions allows the characterisation of local carbon fibre orientations and the presence of fibre voids between single fibre tows. The rotational speed of the probe is 20 revolutions per second with a measuring rate of 20 kHz. The concept, however, is limited to its application of cured composites because the probe movements could harm exposed carbon fibres of dry preforms and therefore damage the tested part beyond repair. In addition, the linear movement of the probe is bound to planar or at least large-radius structures [10].

Heuer presents a high resolution approach to characterise the quality of single fibre tows using a robot guided sensor system. A complex impedance signal, which is influenced by the electric conductivity and permittivity of carbon fibres, is measured and allows the interpretation of fibre tow orientations. When combined with the path data of the robot’s tool centre point and Fourier analysis, a three-dimensional evaluation of the analysed carbon fibre structure can be realised. The novelty of this system is the use of industrial robots that are automatable and, therefore, enable production scenarios with an in-line quality control of semi-finished textiles. This integration type however is characterized by stations on the shop floor that require additional space and lead to increased cycle times. Therefore, the drawbacks of this concept are the limited integrability due to the use of robots and the restricted testability of small component radii because of the probe’s and robot arm’s sizes [9].

The German Aerospace Center (DLR) applies the system as presented above for the detection of fibre misorientations, ondulations and foreign body inclusions in aerospace components [11].

To reduce measurement times, Heuer also presents the possibility to use more than two electric coils in the shape of a coil line array. By increasing the number of pickup coils, the mechanical movement can be replaced by an electric field movement. However, sufficient high switching speeds need to be realised to maintain the Tool Center Point (TCP) speed of the robot. In addition, when testing small part radii, the size of the line array is adverse. Therefore, the advantages of the presented line-array are the capability of non-destructive testing of large radius parts with short measurement times [12].

Koyama’s work aims for the systematic variation of shapes for emitting and pickup coils and how this affects the detectability of various defect types, sizes and positions under laboratory conditions. The focus lies on intentionally inflicted foreign object damage between single fibre layers. It could be shown that signal-noise-ratios can be increased by adapting the relative positions inside half-transmitting sensor arrangements. The arrangements used cannot be applied to half transmitting arrays. However, it leads to the conclusion that mixing circular and rectangular coil cross sections may lead to advantages in design and signal characteristics of eddy current sensors [13].

Cheng’s approach focuses on the research of an electromagnetic model for calculation of eddy currents in CFRP laminate composites. The analytical connection between electrical characteristics and laminate structures are described in numerical models. The models are then used to interpret measurable eddy current signals when testing delaminated carbon fibre laminates that are an issue in cured laminates but however have no importance for semi-finished preforms. Therefore, the applicability of Cheng’s findings to the eddy current characterisation of preforms is limited [14].

Mizukami’s investigations focus on the variation of cross-section shapes and sizes of both emitting and pickup coils and how it affects their sensitivity when testing carbon fibre textiles. However, there is no information given about the interactions with single fibre tow orientations. Lange and Mook showed that a higher sensitivity does not necessarily mean a high directionality of the sensor signal when evaluation fibre tow orientations. Furthermore, the specimen temperature and its effect on the sensor signal are evaluated. It was observed that temperature changes of 30 K lead to a signal damping of up to 2.5% [15].

Salski presents an approach where rigid printed circuit boards (PCB) are used as line arrays that are moved mechanically above the specimen of a cured CFRP. The benefit of using PCBs compared to solenoid coils is that geometrical coil characteristics can be realized with a high reproducibility. In addition, the possibility to use flexible PCBs for this purpose is pointed out, however is not realized experimentally. Flexible PCBs could allow the testing of complex, non-planar surfaces, which may be beneficial when testing components with small radii and intricate geometries. However, no realised prototype is presented [16].

Altogether, it is evident that there are various scientific questions in eddy current testing of CFRP that are covered by the described approaches. However, none of the approaches presented above aims for the application of static eddy current sensors. In the existing research work, the presented approaches where the designed probes are bound to mechanical movement, either rotatory or translational. In addition, only trivial sensor array designs with uniform shapes are applied even though it was shown that proper sensor designs can lead to significant improvement of signal characteristics. The expansion of the state of the art in eddy current testing that is presented in this work uses the half-transmitting principle in a concentric coil array to characterize carbon fibre preforms during their formation.

## 2. Materials and Methods

**Figure 2:**
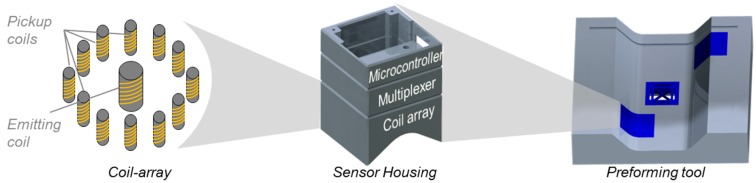
In-process integration of eddy current sensor arrays in preforming tools.

The approach that is presented in this article describes the process integration of static eddy current sensors and the experimental determination of design characteristics. In many industrial and scientific applications, the preforming process during Resin Transfer Moulding is realized with forming tools that have the same geometrical features as the component that is formed. Due to the low mechanical stability of the textile, comparatively low process forces are necessary to shape the semi-finished part. Therefore, the concept includes the substitution of individual tool areas by sensor casings that contain an array of coils, which is capable of testing carbon fibre textiles during preforming. Thus, 100% quality control is enabled, because every preformed part can be tested during preforming without additional measurement stations or processes within the process times. This is a significant benefit for production processes, as it leads to important information about part qualities. This type of quality inspection is a major goal for many producing companies. However, this approach is mainly beneficial for highly automated large volume production systems with predefined regions of interest that need be identified prior to adapting preforming tools with the described sensor concept. Figure 2 illustrates the described concept.

**Figure 3:**
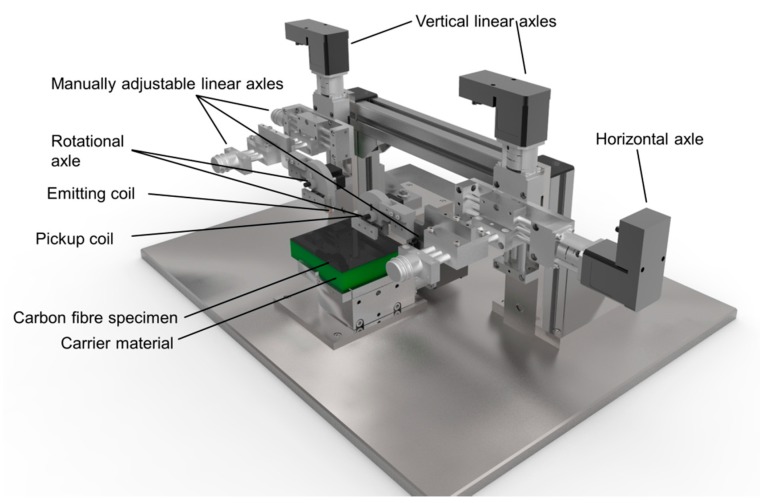
Measurement rig for the determination of optimal sensor designs.

To determine the optimal angular resolutions for the sensor array, different experiments were conducted on a measurement rig that can automatically vary relative positions of half-transmitting coil arrangements (see Figure 3). The goal is to estimate the trade-off between signal quality and the possible geometrical properties that may be necessary to realise complex shaped eddy current sensor arrays. An experimental analysis of the described characteristics is important because both analytical and numerical methods are bound to uncertainties due to assumptions that do not necessarily represent realistic circumstances. In addition, the evaluation of PCB based coils is challenging compared to cylindrical coils. In this context, the relative positions between emitting and pickup coil is varied in lateral, saggital and angular axis, respectively, with linear and rotational mechanical axles. In addition, different types of electric coils can be mounted and their transmission characteristics inspected both with and without the presence of carbon fibre material.

The coils that were used in the presented work are realised as printed circuit boards (PCB), consisting of one emitting coil with a circular cross section with a diameter of 12 mm and 12 turns (see Figure 2). The cross section of each turn is 0.152 mm × 35 × 10⁻^3^ mm. The pickup coil is rectangular with the edge sizes 8 mm × 12 mm and 10 turns. A Digilent Analog Discovery 2 digital oscilloscope with a resolution of 0.37 × 10⁻^3^ V in a range of ±25 V measures the induced voltage in the pickup coil. The emitting coil is excited by a 10 V peak to peak signal with a frequency of 15 MHz. The results that were gained with the measurement rig are subdivided in the transmission characteristics without the presence of carbon fibre specimens as well as with a woven one layer carbon fibre specimen. 

### 2.1. Sensor Characteristics without Carbon Fibre Influence

Due to complex geometries of forming tools, the relative position of emitting and receiving coils may vary. This leads to changes in the transmitting behaviour of the coils, which is limited. Therefore, within the experimental investigations, the described coils are varied in their relative positions using a full factorial experimental design, which leads to 72 single measurements for the characterization of the transmission behaviour for the described sensor type, that were conducted once. The alternating voltage was measured for 2 s with a frequency of 100 MHz. Therefore, it is assumed that system inherent deviations are averaged well for each measurement setup. The analysed system parameters, their variations and how they affect the sensor array design are shown in Table 1.

The coil tilt results from complex, uneven geometry shapes that need to be respected when integrating sensors in non-planar tools. In the presented work, the relative tilt was varied, 0°, 5°, 10° and 15°, and can be described as a rotation between the depicted coordinate systems of the coils, located at their adjacent edges. The defined parameters are transferred to the positions of the rotational axles on the test rig. The variation of the lateral distance is used for the determination of possible sensor layouts that are not circular because multiple receiving coils may be arranged parallel to each other. Therefore, the measurement of the induced voltage as a subject of lateral shifts varied between 0 mm, 5 mm and 10 mm is conducted. The variation of radial distances defines the sensor diameter and is varied from 0 mm edge to edge distance to 25 mm in 5 mm increments. Therefore, the investigations of effects of the described geometry variations can be applied for a general characterization of possible sensor designs.

**Figure 4:**
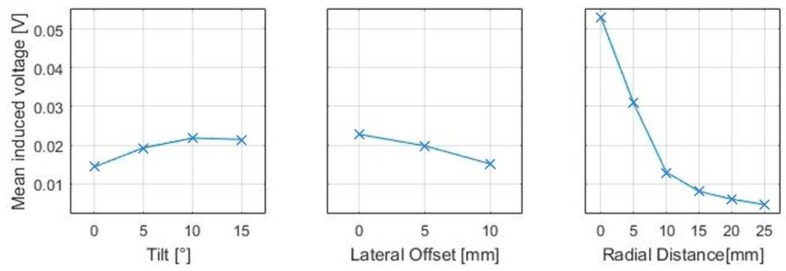
Main effect diagrams of relative positions for PCB based coils.

The results of the systematic variation of the relative positions between emitting and pickup coil and their effect on the measureable voltage can be represented in a main effects diagram where the effects of coil tilt, lateral and radial distances can be seen (Figure 4). It could be shown that an increased coil tilt leads to an increased induced voltage, whereas the lateral offset leads to a decrease of the mean induced voltage. In addition, it is evident that an increased radial distance has the greatest effect on the signal amplitude. These effects can be interpreted by the interactions between the shape of magnetic field, generated by the emitting coil and the position of the receiving coil in relation to it.

Furthermore, the analysis of interactions between these factors has shown that an increased tilt can even lead to an increase of induced voltages. In the context of sensor integration, the presented experimental results can be used for a first estimation about how different sensor designs would affect the measurements prior to investigating the interactions with carbon fibre material. The benefit of this approach is that rather complex formulas and error prone numerical modelling can be avoided in the basic characterisation of the sensor. At the same time, the results can be used for a parametrisation of numerical models.

### 2.2. Sensor Characteristics under Carbon Fibre Influence

**Figure 5:**
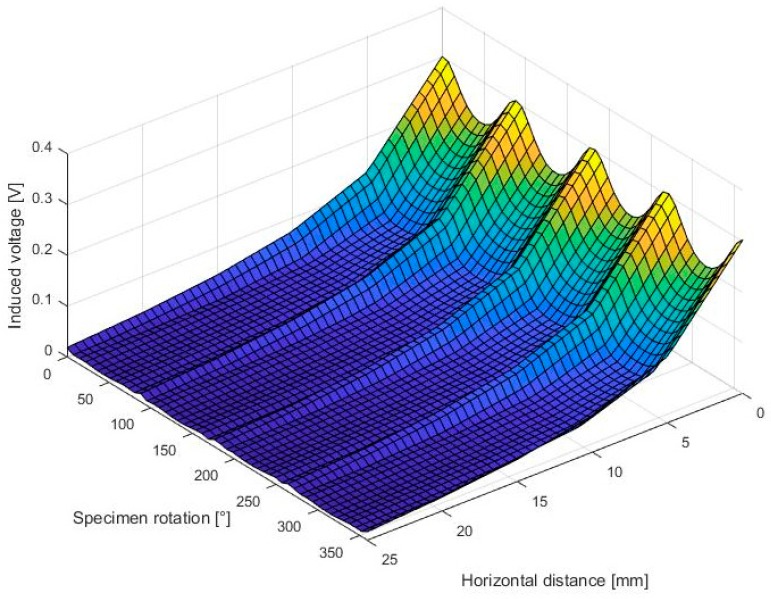
Position sensitive signal characteristics of PCB based sensors when analysing a one-layer woven carbon fibre textile.

In the analysis of the interactions between sensor design and carbon fibre material, a one-layer plain woven carbon fibre fabric with 3000 fibres per roving (Torayca TF300B, Source: bacuplast Faserverbundtechnik GmbH, Germany) was rotated beneath the coil arrangement from 0° to 360° in 5° increments. Simultaneously, the edge to edge distance between emitting and pickup coil was systematically varied from 5 mm to 25 mm in 5 mm increments while measuring the induced voltage. Due to the importance of the horizontal distance in the previously described investigation step, the following results are limited to the effects of this factor. The result can be seen in Figure 5.

The illustrated signal profile can be described as a periodic signal along the specimen rotation axis, which represents the measurable fibre tow orientations in warp and fill directions. The measured peaks along the specimen rotation in the signal courses (0°, 90°, 180° 270°, 360°) indicate the orientation of carbon fibre tows that are located beneath the coil arrangement. As could be expected, the signal amplitude worsens with an increased horizontal coil distance and is comparable to the signal course without the influence of carbon fibre material.

This signal map can be used to determine possible design restrictions, by analysing the gradients at specific points. Evaluation criteria for the assessment of the signal quality are the measured peak-to-peak as well as the mean voltage. The peak-to-peak voltage is calculated by the difference between high and low peaks and needs to be maximized, as well as the mean voltage values. However, both values are decreasing with an increased radial distance, which is detrimental when discussing possible sensor designs such as the circular scheme that is seen in Figure 2. At the same time, possible angular resolutions increase due to the increasing available space. Therefore, a compromise between the signal quality criteria and the possible angular resolution must be found. 

In reference to [17], the %RE can be used to calculate the minimum resolution to differ from different fibre orientations as a criterion for measurement system capability. This value is strongly connected to the peak-to-peak voltage: When measuring values in between the peak-to-peak values, the assignment to a linear characteristic allows the calculation of a specific angle. Therefore, %RE of a measurement system can be calculated using Equation (1) with RE as the absolute resolution that is 0.37 × 10⁻^3^ V and RF as the assumed specified tolerance of 5° [17].

(1) $$\%RE=\;\frac{RE}{RF}\times 100\%\;\begin{array}{c} !\\ \leq \end{array}5\%$$

**Figure 6:**
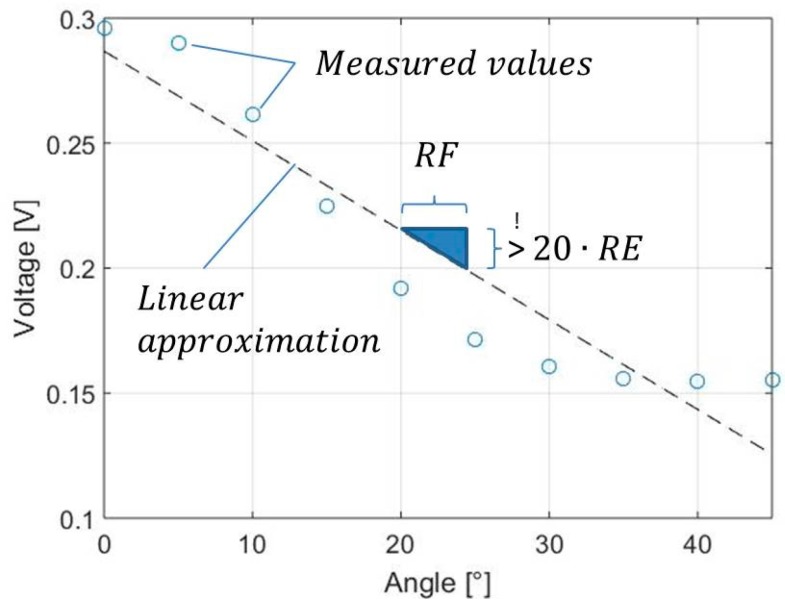
Linearization of orientation specific voltages for the determination of %RE.

For the calculation of %RE, RF needs to be expressed in terms of a measurable voltage instead of an angular production tolerance. Therefore, the measured values that represent fibre tow orientations of 0° to 45° are linearized and RF is expressed as a voltage. The reason why the linearization must take place within the described angle width (0°–45°) results from the possible maximum and minimum values that can theoretically be gained in this measurement setup. For the investigation of unidirectional textiles, the angle width must be adapted to 0°–90° because the location of the minimal measurable value is defined by the main fibre directions. The linear characteristic can be used to calculate RF, which was determined to be 0.066 V. This value shall not be surpassed by the described sensor concept to reliably determine fibre tow orientations with a production tolerance of 5°. This value, however, is only valid for the inspected material type in a planar shape. The linearization that must take place for this purpose is seen in Figure 6. In addition, the meaning of the variables from Equation (1) is shown.

**Figure 7:**
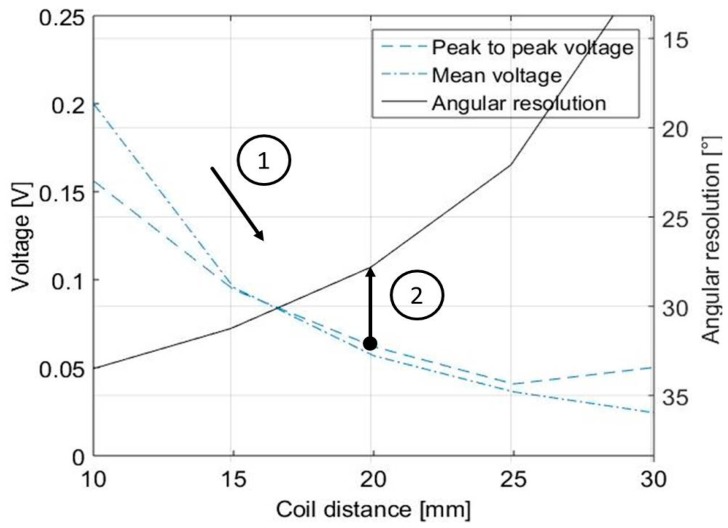
Material- and sensor-specific determination of possible angular resolution.

By using this approach, the difference between measured values at 0° and 45° can be plotted for every tested coil distance, which allows an easier visual interpretation compared to Figure 5, however still includes the same information. At the same time, the diameter of the sensor is given by the distance and sizes of both emitting and receiving coils. When comparing these characteristics to each other, the trade-off that must be found in the design of the sensor is visible. By using the lowest limit of RF that was determined in the first step, the theoretical angular resolution for the circular sensor array can be derived graphically in Figure 7. Thus, the first step that needs to be taken for the determination of possible resolutions includes the determination of the maximum coil distance with regard to the lowest limit of RF and is tagged with “1”. Based on the maximum distance, the angular resolution is determined in “2” with the given coil sizes.

For the discussed sensor and specimen material the resulting angular resolution is approximately 28°. Together with the width of the analysed receiving coils, this value leads to a maximum of 12 receiving coils. This approach can theoretically be applied to any factor that is influencing the signal characteristics in half-transmitting probes. The findings are transferred to a sensor layout that is discussed in the following section.

## 3. Results

### 3.1. Transfer of Results to an Eddy Current Sensor Array

**Figure 8:**
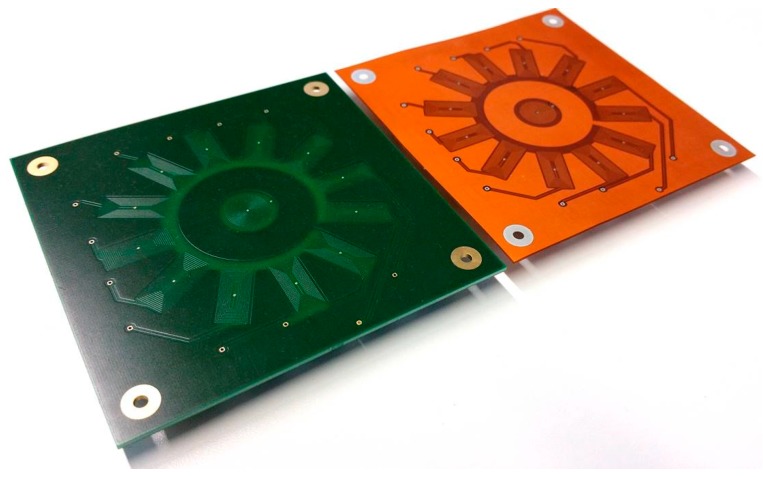
Rigid (**left**); and flexible (**right**) PCB based eddy current sensor array.

The use of a circular emitting coil allows the generation of an isotropic magnetic field, which makes the signals of all pickup coils comparable. The sensor was realized as a rigid version for the testing of planar surfaces in the preforming process and another, flexible version for curved textile surfaces that are prone to wrinkles (see Figure 8). The working principle of the sensor is based on the measurement of induced voltage in each pickup coil that is switched by a multiplexing unit. 

The housing of the sensor is separated into three modules, as is shown schematically in Figure 2. The top module contains an Arduino Mega 2560 Board. It supplies the multiplexing units with power and stores the information about switching routines such as measuring time and switching sequence. The mid module contains all integrated circuits that are necessary to realise the magnetic field movement. The low module is the mechanical interface for the inspected parts and contains the sensor array. It has the same geometry as the tool part, which it substitutes and needs to be adapted from application to application. During the experimental testing, a stereolithography based 3D printer by Formlabs was used to produce redesigned parts. The flexible PCB, which is positioned on the bottom side of the 3D printed part, can be detached and reused after the geometry is redesigned.

### 3.2. Zero Referencing

**Figure 9:**
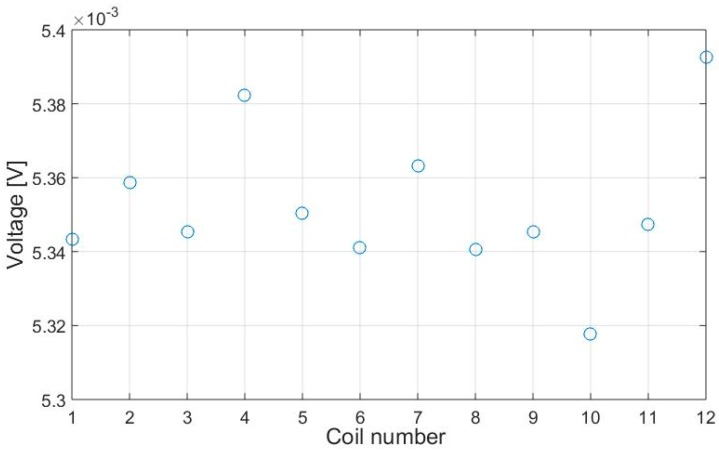
Zero referencing values for the used pickup coils.

Each coil pair has its own signal transmission characteristics due to unavoidable disturbing factors such as the conductor length, the quality of the soldering joint or manufacturing variations. These factors have effects on the electric properties of the sensor, which is why it is to be referenced prior to its application. This is done by executing multiple measurements without being near to carbon fibres. The result consists of the voltages of each coil pair considered as $C_{1}\ldots C_{12}$ that can be seen in Figure 9.

All resulting values are saved and used during the measurement routine to achieve a higher comparability between the pickup coils of the array.

### 3.3. Eddy Current Array Measurement Results

After the zero referencing, the sensor is tested with a material specimen that consists of a material of woven carbon fibre fabric with the fibre tow orientations 0°/90°. For each measurement, altogether twelve single measurement values $U_{1}\ldots U_{12}$ are obtained by the respective twelve receiving coils. To demonstrate the capability of the sensor the specimen was rotated by 10°-steps in relation to the sensor. The resulting values were processed with the reference data to obtain a resulting vector $\vec{Y}$.

(2) Y→=(Y1Y2…Y12)= (U1C1U2C2…U12C12)

Each value of the resulting vector can is then plotted in a polar diagram, in order to achieve a figure that is comfortable to interpret visually. The values $Y_{1}\ldots Y_{12}$ represent the gain of each coil, which is interpolated via discrete Fourier transformation to interpolate values in between the available single measurement values. The benefit of using this method is that values can be interpolated in between the measured values under consideration of periodic signal characteristics. This leads to a higher reliability in the determination of fibre tow orientations. Table 2 contains the measurement results that were gained during the tests of the developed sensor.

## 4. Discussion

It can be seen that the difference between the nominal rotation angle and the measured angle by the sensor does not necessarily depend on each other. Therefore, it can be assumed that the angular resolution of the sensor has a subordinate role in comparison to the sensor sensitivity. However, the shape and peak amplitudes of the measured signals vary significantly between the cases where fibre tow orientations align with the coil axis (see rotation step 0° and 30°) and where the values are interpolated (see rotation step 10° and 20°). The differences in signal shapes offer the possibility to be classified, e.g., by using tools of pattern recognition. However, it can be said that the basic shape of the signal strongly resembles the signals that can be obtained by mechanically moving a pairwise sensor above the specimen. This allows the reduction of measurement times in the production of carbon fibre preforms and at the same time increasing the quality information. Due to the similarity of the signal shapes, it can be assumed that other material types (unidirectional laminates or other weave patterns, such as twill weaves) that were already inspected in the state of the art can also be inspected with the described system. However, the angular resolution needs to be taken in consideration for each different material structure because there is a minimum amount of receiving coils that shall not be surpassed to reconstruct a reliable signal shape with e.g., more than four main peaks. The experimental estimation of the measurement system uncertainty and process uncertainties according to [17] will be conducted in further works. 

## 5. Conclusions

The presented work describes an approach for the development of static eddy current arrays that can be integrated in the preforming step of Resin Transfer Moulding. This allows the reduction of measurement times during the quality inspection of carbon fibre preforms. An experimental approach on the determination of possible angular resolutions with respect to the achievable signal quality is discussed. It could be shown that basic signal characteristics such as the peak-to-peak voltages allow the estimation of best possible sensor diameters and respective sensor resolutions. Furthermore, a zero referencing routine is described and how the generated values are utilised during the application of the sensor. A material specimen was created for first testing purposes of the eddy current sensor array that need to be expanded in further investigations. Especially, the capability of the measurement system needs to be characterised to enable 100% quality control. However, it was shown that fibre tow orientations of the specimen can be determined with a high reliability by using discrete Fourier transformation. Further work will focus on the experimental determination of design specifications as a subject to part geometries for flexible sensor arrays.

## Figures and Tables

**Table 1:** Relative coil positions.

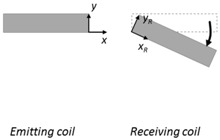	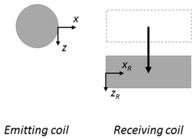	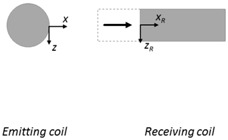
Tilt	Lateral distance	Radial distance

**Table 2:** Eddy current sensor array measurements.

Rotation Step: 0°		Rotation Step: 10°		Rotation Step: 20°		Rotation Step: 30°	
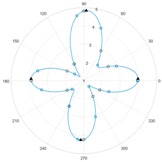		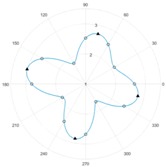		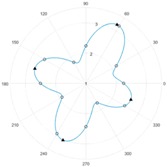		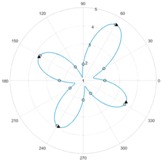	
**Nominal Angle**	**Measured Angle**	**Nominal Angle**	**Measured Angle**	**Nominal Angle**	**Measured Angle**	**Nominal Angle**	**Measured Angle**
0°	2°	350°	347°	340°	340°	330°	333°
90°	88°	80°	76°	70°	62°	60°	59°
180°	178°	170°	166°	160°	164°	150°	152°
270°	267°	260°	259°	250°	248°	240°	242°
**Mean Error:**							
1.25°		3°		1.5°		−1.5°	
